# Neurodevelopmental Disorder with Dystonia and Chorea Linked to De Novo Variants in the Splicing Regulator *SRRM4*

**DOI:** 10.1002/mds.70297

**Published:** 2026-04-09

**Authors:** Philip Harrer, Volker Kittke, Alice Saparov, Alexej Knaus, Shimriet Zeidler, Rachel Schot, Florian Kraft, Matthias Begemann, Suzanna Koudijs, Ugo Sorrentino, Chen Zhao, Ivana Dzinovic, Martin Pavlov, Elisabeth Graf, Antonia M. Stehr, Peter M. Krawitz, Christian Wilhelm, Saskia Biskup, Fahd Alsalloum, Steffen Berweck, Juliane Winkelmann, Konrad Oexle, Ingo Kurth, G. Christoph Korenke, Michael Zech

**Affiliations:** ^1^ Institute of Human Genetics, School of Medicine and Health Technical University of Munich Munich Germany; ^2^ Institute of Neurogenomics Helmholtz Zentrum München Munich Germany; ^3^ DZPG, Deutsches Zentrum Für Psychische Gesundheit Munich Germany; ^4^ Helmholtz Association‐Munich School for Data Science (MUDS) Munich Germany; ^5^ Institute of Genomics Statistics and Bioinformatics, School of Medicine, University Hospital Bonn University of Bonn Bonn Germany; ^6^ Department of Clinical Genetics Erasmus MC, University Medical Center Rotterdam Rotterdam The Netherlands; ^7^ Center for Human Genetics and Genomic Medicine, Faculty of Medicine RWTH Aachen University Aachen Germany; ^8^ Neurology Department Erasmus MC, University Medical Center Rotterdam Rotterdam The Netherlands; ^9^ CeGaT und Praxis für Humangenetik Tübingen Tübingen Germany; ^10^ Specialist Centre for Paediatric Neurology, Neurorehabilitation and Epileptology Schoen Clinic Vogtareuth Vogtareuth Germany; ^11^ Center of child neurology, developmental medicine and rehabilitation, Children's Hospital of Eastern Switzerland St. Gallen Switzerland; ^12^ Munich Cluster for Systems Neurology, SyNergy Munich Germany; ^13^ Neurogenetic Systems Analysis Group, Institute of Neurogenomics Helmholtz Munich Neuherberg Germany; ^14^ Department of Neuropaediatric and Metabolic Diseases University Children's Hospital Oldenburg Oldenburg Germany; ^15^ Institute for Advanced Study Technical University of Munich Garching Germany

**Keywords:** alternative splicing, chorea, dystonia, genome editing, genomics, neurodevelopmental disorder, transcriptomics

## Abstract

**Background:**

*SRRM4* is an exclusively neural‐expressed splicing‐factor gene not yet associated with a monogenic condition.

**Objective:**

We sought to delineate movement disorders caused by *SRRM4* variants. De novo splice‐donor‐site variants at position +2 of intron 5 of *SRRM4* (c.464+2T>C, c.464+2T>A) occurred in three unrelated patients with dystonia and chorea. We present detailed phenotypic information on these individuals and characterize the effect of the splice‐site alteration.

**Methods:**

Exome and genome sequencing were used to identify *SRRM4* variants. To assess the consequence of a mutant +2 residue at the affected splice donor of *SRRM4*, we performed transcriptomic analyses using short‐read and long‐read RNA‐sequencing in patient fibroblasts in which *SRRM4* expression was induced by genome editing.

**Results:**

Clinical presentations were characterized by infantile combined dystonic and choreatic syndromes or chorea‐predominant disease. Studies in *SRRM4* expression‐activated cells revealed two variant‐specific *SRRM4*‐mRNA isoforms including one that was characterized by a 69‐nucleotide in‐frame insertion without creation of a premature termination codon, suggestive of a mechanism other than loss‐of‐function. Additionally, we uncovered altered splicing patterns of known *SRRM4* downstream mRNA‐substrates in patient cells compared to *SRRM4* expression‐activated control fibroblasts, such as a conserved *AP1S2* microexon. *AP1S2* is linked to a monogenic syndrome with abnormal movements and missplicing of its microexon is a well‐established outcome in neural models of *SRRM4* disruption.

**Conclusions:**

We conclude that the patients' phenotypes are caused by a previously undiagnosed *SRRM4*‐related disorder, offering a basis for improved understanding of mechanistic convergence in genetic movement disorders and potential therapeutic targeting of the misregulated splicing events. © 2026 The Author(s). *Movement Disorders* published by Wiley Periodicals LLC on behalf of International Parkinson and Movement Disorder Society.

Early‐onset movement disorders are disabling conditions associated with wide‐ranging motor and non‐motor features.^1^ Although a few hundred genes have been causally related to these entities,^1^ much of their etiological bases remains to be discovered. Alterations of genes encoding factors of the alternative splicing (AS) machinery are increasingly recognized as causes of pediatric neurodevelopmental and neurodegenerative syndromes that can manifest with abnormal movements.^2^, ^3^, ^4^, ^5^ AS expands the diversity of cellular mRNA transcripts at different developmental stages, a critical mechanism for tissue‐identity acquisition and organ maintenance.^6^ Nevertheless, evidence for the involvement of AS regulators in specific genetic movement disorders is still limited. *SRRM4* codes for the neural‐specific serine/arginine (SR) repetitive matrix protein 4 (SRRM4, also known as nSR100), which belongs to the class of SR‐related proteins and controls AS programs with essential roles in neurophysiology.^7^ SR‐related protein family members share the ability to interact with mRNA and additional protein components participating in regulated splicing.^7^, ^8^ SRRM4 is a potent activator of alternative cassette exon inclusion into the mRNAs of genes involved in neurogenesis.^7^, ^9^, ^10^ Disruption of SRRM4 produced developmental deficits in model organisms, including impaired neurite outgrowth and changes in mouse brain morphology.^7^, ^9^, ^10^ Transcriptome‐wide profiling in murine neural cells revealed that SRRM4 downregulation resulted in the exclusion of a well‐defined set of conserved exons, whereas induced expression of SRRM4 in non‐neural cells led to the splicing‐in of the majority of these exons.^10^ Among the SRRM4‐dependent targets are exons of genes that have established links to neurodevelopmental and movement disorders.^10^ For example, *SRRM4* was shown to regulate AS of *AP1S2*, associated with Pettigrew syndrome (Online Mendelian Inheritance in Man [OMIM]: 304340); *ATP6V0A1*, associated with developmental and epileptic encephalopathy‐104 (OMIM: 619970); *DCTN1*, associated with Perry syndrome (OMIM: 168605); *FRYL*, associated with Pan‐Chung‐Bellen syndrome (OMIM: 621049); *MADD*, associated with neurodevelopmental disorder with dysmorphic facies, impaired speech, and hypotonia (OMIM: 619005); *SGCE*, associated with myoclonus‐dystonia (OMIM: 159900); *SPTAN1*, associated with autosomal dominant spastic paraplegia‐91 with or without cerebellar ataxia (OMIM: 620538) as well as developmental and epileptic encephalopathy‐5 (OMIM: 613477); and *TAF1*, associated with X‐linked dystonia‐parkinsonism (OMIM: 314250). For a comprehensive list of previously determined *SRRM4*‐dependent genes, see Raj et al^10^ and Capponi et al,^11^ and for their associations to monogenic disorders, see the OMIM database.^12^ Despite these observations, *SRRM4* remains unassociated with human monogenic traits and patient‐derived variant alleles in this gene have not yet been studied. A better understanding of the mechanistic networks of disease genes connected by AS‐machinery defects and implicated in similar phenotypes can provide insights into pathophysiology and targets for treatment.^4^, ^5^, ^13^ Here, we used an integrative approach using case‐matching and phenotype analyses, genomics, activation of SRRM4 expression in patient and control fibroblasts by CRISPR/Cas9 technology, as well as short‐read and long‐read RNA sequencing (sr/lrRNA‐seq) to build support for a causal role for *SRRM4* in monogenic disease. We present detailed clinical information on three unrelated children with de novo splice‐donor‐site variants at position +2 of intron 5 of *SRRM4* who have overlapping, albeit individually variable, strikingly severe movement disorders and neurodevelopmental comorbidities. Creating new frame‐preserving *SRRM4*‐mRNA isoforms, the mutant splice‐donor site of intron 5 was associated with misregulation of AS of previously described^10^ exon targets of SRRM4. An identified pronounced change in inclusion level of a key functional exon of SRRM4's known neural splicing substrate *AP1S2*,^10^, ^14^ a gene linked to a rare neurodevelopmental disorder with dyskinesia,^12^, ^15^ may offer a plausible explanation for clinical abnormalities of the reported patients.

## Subjects and Methods

### Patients and Genomic Analyses

**FIG. 1:**
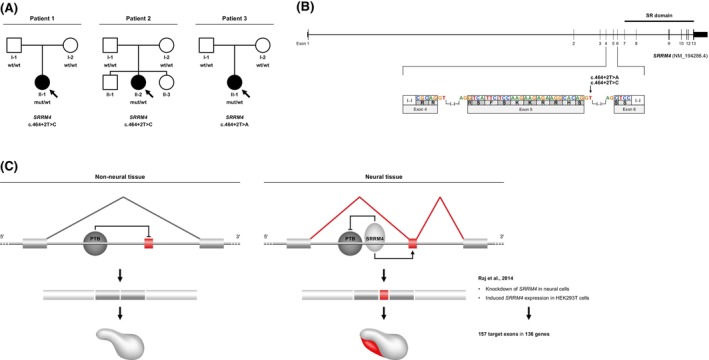
De novo *SRRM4* canonical splice‐site variants identified in three unrelated patients. (**A**) Pedigrees of patients 1, 2, and 3, with indications of *SRRM4* genotypes. Mutant/wild‐type (mut/wt) denotes a heterozygous carrier status for a *SRRM4* variant, and wt/wt denotes a homozygous wild‐type allele status (parents in the three pedigrees). Arrows indicate the patients. (**B**) Diagram of the *SRRM4* transcript NM_194286.4. The 13 exons are represented by black rectangles. A close‐up view of exon 5 and the canonical splice‐donor site affected by the patient‐specific c.464+2T>C and c.464+2T>A variants is shown. The known functional serine/arginine (SR)‐rich domain of the encoded protein is drawn above the transcript. (**C**) A cartoon of the SRRM4 protein (also known as nSR100) in association with the regulatory factor PTB (drawn without excessive details).^10^ SRRM4 controls alternative exon inclusion events in neural cells.^7^, ^10^ Induced *SRRM4* expression in non‐neural HEK293T cells has previously been shown to result in the splicing‐in of 157 neural target exons of *SRRM4*.^10^ [Color figure can be viewed at wileyonlinelibrary.com]

*SRRM4* de novo variants in three unrelated individuals with syndromic movement disorders were determined independently as compelling candidates for disease causation by participating investigators (Fig. 1A,B). Multiple routine diagnostic evaluations guided by senior pediatric neurologists and clinical geneticists had been unrevealing for each patient, prompting further genomic investigations on a research basis. Samples and data from the patients and their biological parents were collected after obtaining informed consent. All associated explorations were conducted in accordance with the Declaration of Helsinki and the local institutional review‐board requirements. Patient 1 was part of a whole‐genome sequencing (WGS) study on multiyear‐undiagnosed dystonia‐affected families.^16^ Illumina short‐read WGS for patient 1 followed a trio‐based approach, as detailed earlier.^16^ Patient 2 underwent repeated trio whole‐exome sequencing (WES) using standard 100‐bp paired‐end protocols in three parallel programs investigating the molecular origins of rare diseases,^17^, ^18^, ^19^ which used their respective clinically oriented bioinformatic pipelines for gene prioritization. Matchmaking for patients 1 and 2 was achieved through personal communication in local expert networks for unexplained monogenic conditions.^16^, ^19^ Patient 3, who had undergone trio WGS, was connected to this study via GeneMatcher.^20^

### Fibroblast Culture and Induced SRRM4 Expression

Because the candidate *SRRM4* was exclusively expressed in neural tissues^7^, ^21^ and undetectable in easily accessible patient samples, we chose to apply CRISPR activation (CRISPRa),^22^, ^23^, ^24^ a targeted gene induction strategy, to test the impact of the identified mutant splice‐donor site on *SRRM4* pre‐mRNA splicing as well as downstream AS outcomes. Fibroblast cultures were established from skin‐biopsy specimens of patient 1 and two control subjects. CRISPRa for *SRRM4* was performed as previously described,^24^ with minor modifications. In brief, we used lentiviral vectors for transformation of the CRISPRa system, expressing a specific guide RNA directed to the promoter region of the canonical isoform of *SRRM4* (NM_194286.4; sg*SRRM4*). To obtain non‐*SRRM4*‐activated negative controls, additional cultures were transfected with vectors containing non‐targeting RNAs (sgNT). Cells were transformed in five biological replicates for each fibroblast line and condition (sg*SRRM4*, sgNT) and cultured for 7 days under antibiotic selection before RNA isolation. Activation of *SRRM4* was verified by reverse transcription (RT) polymerase chain reaction (PCR) with primers spanning exons 2–7; see Supplementary Methods for additional details on CRISPRa experimental procedures.

### srRNA‐Seq

Total RNA was extracted from CRISPRa‐competent fibroblasts using the RNeasy mini kit (QIAGEN) and quality‐controlled using Agilent's Bioanalyzer. Stranded poly(A)‐selected Illumina mRNA libraries were prepared (TruSeq) and sequenced on a NovaSeq6000 according to the manufacturer's specifications.^16^ A minimum of 50 million 150‐bp reads were collected per sample.

### srRNA‐Seq Bioinformatics

Raw data from srRNA‐seq were processed on an in‐house‐established infrastructure for RNA studies.^16^, ^25^ Reads were mapped to hg19/GRCh37 with STAR‐aligner, and visualizations were done with the Integrative Genomics Viewer (IGV).^26^ For differential expression analyses, read counts were calculated and compared in DESeq2.^27^ To assess AS profiles in srRNA‐seq data transcriptome‐wide, we used rMATS‐turbo.^28^ Binary Alignment Map(BAM) files created from the read alignments of pooled biological replicates were used as input. Previously, knock‐in of *SRRM4* in a HEK293T line has been shown to promote the inclusion of 157 *SRRM4*‐dependent neural exons in 136 genes (Fig. 1C), highlighting that induced expression of this gene in non‐neural human cells reproduces many AS events of biological relevance.^10^ Therefore, we first ran rMATS‐turbo to identify genes that underwent significantly increased exon splicing‐in on *SRRM4* activation in our control fibroblast lines. In the second step, we determined differences in the amounts of exon inclusion in the fibroblast model‐derived target genes between *SRRM4*‐activated fibroblasts from patient 1 and the controls using rMATS‐turbo. We used a multiple‐testing corrected *P*‐value (false discovery rate [FDR]) <0.05 and an exon‐inclusion level difference >0.05 as thresholds in rMATS‐turbo.

### lrRNA‐Seq

To optimize the visualized analysis of *SRRM4* variant‐specific mRNA isoforms and downstream AS events, we additionally performed lrRNA‐seq on an Oxford Nanopore Technologies (ONT) platform^29^ for *SRRM4*‐activated fibroblasts from patient 1 and one control. ONT sequencing followed a recently described workflow.^29^ Libraries were generated with the complementary DNA‐PCR Barcoding Kit and sequenced using PromethION.

## Results

### Clinical Analyses

Patient 1, an 11‐year‐old girl, was evaluated for infantile‐onset generalized dystonia, chorea, developmental delay, speech impairment, and epilepsy. She was the only child of healthy, non‐consanguineous parents of European origin (Fig. 1A; Table 1) She was born without complications and was noted to have delayed motor milestones at 6 months of age. She walked first steps at 18 months, but never acquired expressive language. At the age of 20 months, she developed dystonic lower‐limb and truncal posturing, followed by the manifestation of multiple hyperkinetic symptoms including choreatic and athetotic movements in all four extremities. Neurodevelopmental impairment continued in childhood, with profound communication and social difficulties as well as signs of cognitive dysfunction. Verbal comprehension was partially intact. She experienced her first tonic–clonic seizure at age 5 years, and was subsequently diagnosed with generalized epilepsy and atypical absences. A recent neurological examination documented dystonia in the limbs and neck, cranial dystonia, intermittent opisthotonic trunk extension combined with jerky trunk movements, and arm choreoathetotic movements. There was a prominent broad stance while standing with marked imbalance, reflecting a potential truncal ataxic component as well. However, significant limb incoordination or dysdiadochokinesia were not observed (Video 1).

**Table 1:** Summary of SRRM4 variant findings and the associated clinical features of patients 1, 2, and 3

	Patient 1	Patient 2	Patient 3
*SRRM4* variant finding			
cDNA annotation	NM_194286.4: c.464+2T>C	NM_194286.4: c.464+2T>C	NM_194286.4: c.464+2T>A
Variant type	Canonical splice site	Canonical splice site	Canonical splice site
Zygosity	Heterozygous	Heterozygous	Heterozygous
Inheritance	De novo	De novo	De novo
Testing method(s)	Trio WGS (after unrevealing trio WES, PMID: 39937650)	Trio WES (repeated)	Trio WGS
Count in gnomAD v4.1 and in‐house control datasets	Not observed	Not observed	Not observed
In silico predictions	CADD: 32, SpliceAI: donor loss: 0.66; donor gain: 0.29	CADD: 32, SpliceAI: donor loss: 0.66; donor gain: 0.29	CADD: 34, SpliceAI: donor loss: 0.99; donor gain: 0.40
ACMG classification	Likely pathogenic (PS2, PM2, PM4, PP3)	Likely pathogenic (PS2, PM2, PM4, PP3)	Likely pathogenic (PS2, PM2, PP3)
Phenotypic characteristics			
Sex	Female	Female	Female
Origin	European (German)	European (German)	European (Dutch)
Age at last examination	11 yr	16 yr	1 yr 5 mo
Motor development	Delayed	Delayed	Delayed
Language	Absent speech, receptive language partially intact	Absent speech	No speech at last assessment
Intellectual abilities	Impaired	Impaired	Too young to assess
Seizures	Generalized epilepsy, atypical absences	One tonic–clonic seizure during febrile illness	Focal seizures
Dystonia; onset	Generalized (continuous), 20 mo	Generalized/dystonic crises, 11 mo	No
Other movement disorders	Chorea, athetosis	Chorea, athetosis	Chorea (continuous, onset 6 m)
Brain MRI	Normal	Normal	Normal
Other	–	Enterovirus infection with secondary worsening in neurological function, dysphagia	Hypotonia and developmental stagnation at age 6 mon after febrile illness, facial features: long philtrum, upslanting palpebral fissures, synophrys

Prominent common characteristics include: *SRRM4* variant at exact the same position (de novo status in each patient), developmental delay, absent speech, early‐onset hyperkinetic movement disorder.

Abbreviations: ACMG, American College of Medical Genetics and Genomics; CADD, Combined Annotation Dependent Depletion; cDNA, complementary DNA; gnomAD, The Genome Aggregation Database; mo, months; MRI, magnetic resonance imaging; WES, whole‐exome sequencing; WGS, whole‐genome sequencing; yr, years.

**Video 1:** Patient 1 carrying the recurrent *SRRM4* de novo c.464+2T>C variant presenting at the age of 9 years with mild limb dystonia, prominent craniocervical dystonia, intermittent opisthotonic trunk extension, and arm choreoathetotic movements. Marked imbalance while standing was evident, associated with jerky trunk movement and a potential component of truncal ataxia. There was no significant limb incoordination, although most purposeful movements were impaired by the hyperkinesias.

Patient 2 was a 16‐year‐old girl who was initially seen by neuropediatric specialists because of developmental‐delay concerns and later followed for status dystonicus and therapy‐refractory dyskinesia. She was born full term to healthy European‐descent parents (Fig. 1A; Table 1). Following a hypotonic period, she started to express abnormal hyperkinetic movements, especially chorea associated with axial‐predominant dystonia, by the age of 11 months (Video 2, segment A). She had severe articulation deficits and her intellectual level was globally below average. Since the age of 8 years, she developed recurrent dystonic crises, which were incompletely resolved by therapy with intrathecal baclofen and other relaxants. These severe acute motor exacerbation episodes presented in the form of dystonic storm phenotypes^30^ (see Video 2, segment B, for demonstration of an acute dystonic exacerbation [patient 2 at the age of 10 years]). Over time, she also presented one tonic–clonic epileptic seizure and persistent worsening in motor functioning with swallowing problems after an enterovirus infection. During her last examination, she exhibited generalized dystonia, distal‐arm athetosis, and chorea with gait dysfunction requiring assistance, absent speech, and dysphagia.

**Video 2:** Segment A: patient 2 carrying the recurrent *SRRM4* de novo c.464+2T>C variant presenting at the age of 21 months with mixed hyperkinetic, mostly choreatic movements. Segment B: patient 2 presenting at the age of 10 years with a severe episode of generalized dystonic movements. The episode represented a dystonic storm phenotype, re‐occurring in the patient during episodes of external stressors such as infections.

Patient 3, a 1‐year‐old girl of European origin, was evaluated for a range of symptoms. She had delayed development noted at 5 months of age because of poor head control. She was increasingly hypotonic since a febrile illness at age 6 months. Continuous abnormal movements appeared soon after, classified as chorea by neuropediatric specialists. A focal seizure was suspected at the same age (6–8 months) that reoccurred in the following months, and she was diagnosed with epilepsy. The latest evaluation at age 1 years and 5 months showed generalized choreatic movements, developmental delay with no spontaneous speech, and minor dysmorphic features (Table 1).

All patients had normal brain magnetic resonance imaging and comprehensive diagnostic screening of hundreds of known disease genes related to the patients' symptoms^12^ did not uncover causative variants. Further clinical characteristics for the patients are summarized in Table 1.

### SRRM4 Variants

**FIG. 2:**
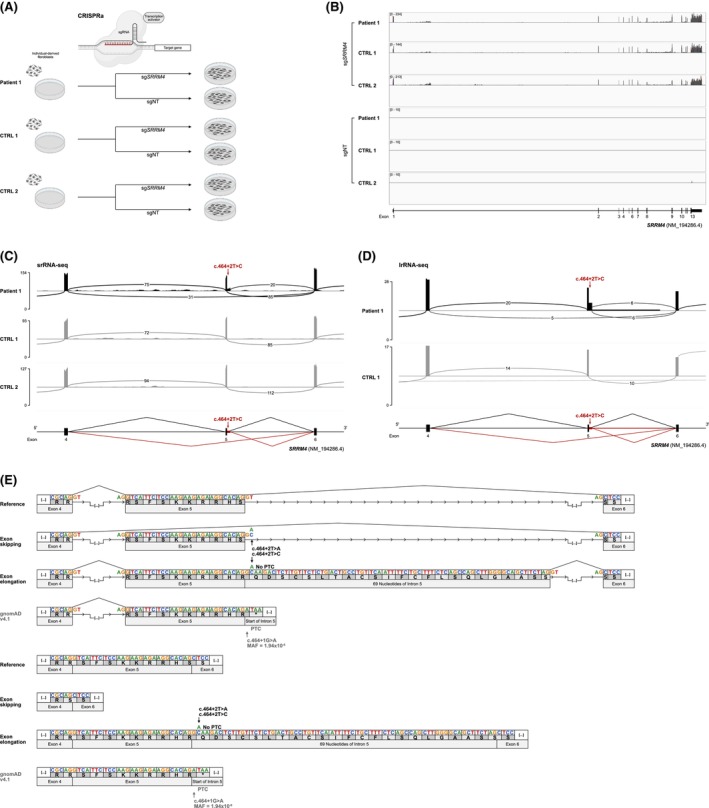
Activation of *SRRM4* expression in fibroblast cell lines and effect of a mutant +2 residue of the splice‐donor site of intron 5 on *SRRM4* pre‐mRNA splicing. (**A**) Schematic representation of the CRISPR activation (CRISPRa) approach used to induce the expression of *SRRM4* in fibroblast cell lines from patient 1 and two independent control (CTRL) subjects (CTRL 1, CTRL 2). A specific guide RNA directed to the promoter region of *SRRM4* (NM_194286.4; sg*SRRM4*) was used. Non‐targeting RNAs (sgNT) served as negative control. (**B**) Representative visualization of short‐read RNA sequencing (srRNA‐seq) data demonstrating robust *SRRM4* expression in fibroblast cell lines from patient 1 and the CTRL subjects (CTRL 1, CTRL 2) following CRISPRa‐based treatment with sg*SRRM4*. No *SRRM4* expression was detected in fibroblasts following CRISPRa‐based treatment with sgNT. (**C**,**D**) Representative sashimi plots established from srRNA‐seq data (C) and long‐read RNA sequencing (lrRNA‐seq) data (**D**) generated for CRISPRa‐competent (*sgSRRM4*) fibroblast cell lines from patient 1 and the CTRL subjects (CTRL 1, CTRL 2). Mutation of the +2 residue of the splice‐donor site of intron 5 of *SRRM4* was associated with skipping of exon 5 (~27% of reads, 25/91, on average in srRNA‐seq data; ~29% of reads, 5/17, in lrRNA‐seq data) as well as elongation of exon 5 (~10% of reads, 9/91, on average in srRNA‐seq data; ~35% of reads, 6/17, in lrRNA‐seq data). The mutation consequences on *SRRM4* pre‐mRNA splicing are highlighted in the schematic drawing (bottom panels). Additional biological replicates are shown in Fig. S2. (**E**) Schematic depiction of the mutant *SRRM4*‐mRNA isoforms associated with a mutant +2 residue of the splice‐donor site of intron 5 of *SRRM4*. The events were expected to be frame‐preserving. In the isoform with the 69‐nucleotide exon‐5 elongation, c.464+2T>C and c.464+2T>A would not be predicted to result in the introduction of a premature termination codon (PTC); in contrast, the presence of a PTC would be expected for a c.464+1G>A variant and other nearby intronic variants listed in gnomAD v4.1 (for details, see Fig. S4). [Color figure can be viewed at wileyonlinelibrary.com]

A recurrent, heterozygous de novo *SRRM4* canonical splice‐site variant located within the donor site of intron 5, NM_194286.4: c.464+2T>C was prioritized by trio WGS^16^ in patient 1 and by trio WES in patient 2 (Fig. 1B). In patient 3, trio WGS discovered a de novo T>A substitution of the same +2 splice‐donor site of *SRRM4*: NM_194286.4: c.464+2T>A. The variants were absent from the Genome Aggregation Database (gnomAD)‐v4.1 and from the in‐house reference datasets of five institutions involved, totaling more than 1.5 million control alleles. In silico algorithms predicted that the variants would be functionally relevant (Table 1). We evaluated *SRRM4* mRNA transcripts in *SRRM4*‐activated fibroblasts derived from patient 1 and two control subjects to understand the effect of a mutant +2 residue of the splice‐donor site of intron 5 (Fig. 2A–E). RT‐PCR analysis showed only the expected *SRRM4* product amplified from control cells, whereas three different amplicons were present in the patient sample (Fig. S1). Visualization of srRNA‐seq data with IGV^26^ confirmed the presence of two mutant *SRRM4*‐mRNA isoforms in addition to the canonically spliced transcript in patient 1's fibroblasts (Fig. 2C and Fig. S2): an isoform lacking exon 5 (NM_194286.4:r.438_464del; ~27% on average in srRNA‐seq); and an isoform containing a 69‐nucleotide elongation of exon 5, created by a cryptic splice donor (NM_194286.4:r.464_465ins[gc;464+3_464+69]; ~10% on average in srRNA‐seq). In lrRNA‐seq data, the latter isoform was observed in approximately 35% (6/17 reads) of patient *SRRM4* transcripts (Fig. 2D). As both aberrantly spliced isoforms resulted in preserved coding frames, *SRRM4* transcripts in the patient were not expected to be degraded by nonsense‐mediated decay (Fig. 2E). In agreement, there was no decreased *SRRM4*‐mRNA expression in srRNA‐seq data from patient 1's cells compared to controls. Rather, we found higher *SRRM4*‐mRNA levels in the patient (fold change = 1.45; *P*_adj_ = 4.65 × 10^−9^; DESeq2) (Fig. S3), which could reflect altered transcript stability in the context of in‐frame mRNA‐length changes or other compensatory mechanisms such as upregulation of the wild‐type allele.^31^ GnomAD‐v4.1 lists a c.464+1G>A substitution with extremely low frequency (1.94 × 10^−6^), located at the same splice site as the patient‐specific variants; inspection of the srRNA‐seq data revealed that c.464+2T>C predictably caused the loss of a premature termination codon that would otherwise be present in the elongated mRNA isoform. The same consequence was expected for c.464+2T>A, but not for c.464+1G>A and other nearby gnomAD‐v4.1 variants (Fig. 2E and Fig. S4). Finally, we modeled the predicted mutant proteins associated with patient 1's c.464+2T>C variant (NP_919262.2:p.(Arg146_His154del); p.(His154_Ser155insXaa [23])) (Fig. S5). The in‐frame deletion/insertion variants were not predicted to cause structural changes of a known functional domain of SRRM4.

### Downstream Effect on Splicing Substrates

**FIG. 3:**
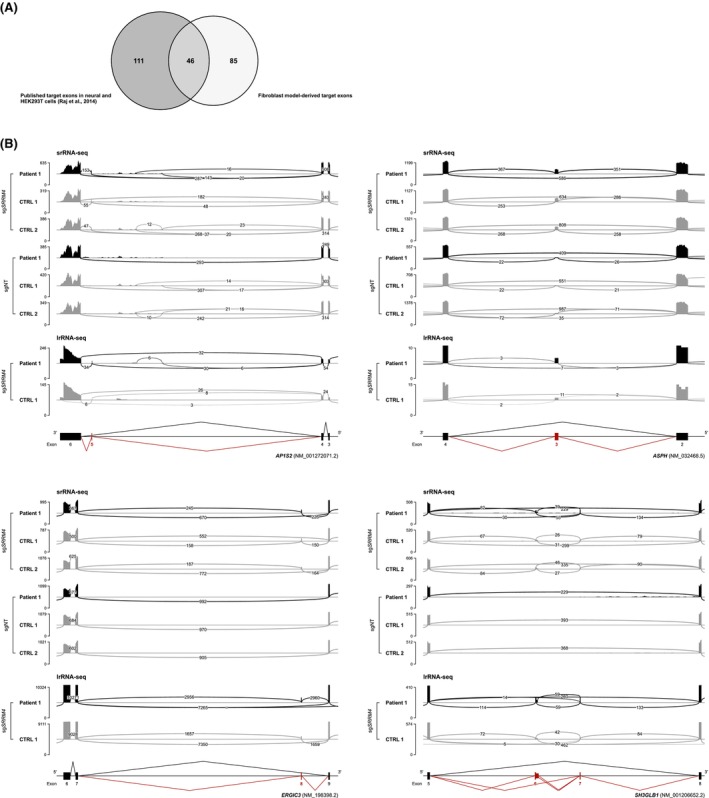
Analysis of changes in alternative exon inclusion in mutant fibroblast cells expressing *SRRM4*. (**A**) Venn diagram illustrating the overlap in alternative exon‐inclusion events between *SRRM4*‐activated control fibroblast cell lines and previously reported *SRRM4* expression‐modified neural and HEK293T cell models.^10^ A total of 157 *SRRM4*‐dependent exons in 136 genes have previously been defined as a set of high‐confidence targets of *SRRM4* in neural and HEK293T cells.^10^ To identify genes with increased exon splicing‐in related to *SRRM4* in our CRISPR activation (CRISPRa)‐competent fibroblast model, we performed rMATS‐turbo^28^ analysis on short‐read RNA sequencing (srRNA‐seq) data generated for fibroblast cell lines from the two control subjects (CTRL 1, CTRL 2) in which the expression of *SRRM4* was induced (CRISPRa‐based treatment with sg*SRRM4*) or not induced (CRISPRa‐based treatment with sgNT). (**B**) Sashimi plots established from srRNA‐seq and long‐read RNA sequencing (lrRNA‐seq) data demonstrating representative changes in alternative exon‐inclusion events in patient 1 carrying the recurrent *SRRM4* c.464+2T>C variant versus the two control (CTRL) subjects (CTRL 1, CTRL 2). rMATS‐turbo analysis^28^ was performed on srRNA‐seq data generated for *SRRM4*‐activated fibroblast cell lines from patient 1 as well as CTRL 1 and CTRL 2. The analysis focused on 111 pre‐defined genes with increased exon‐inclusion events detected in the *SRRM4*‐activated control‐subject fibroblasts. Compared to controls, *AP1S2*, *ASPH*, *ERGIC3*, and *SH3GLB1* showed significantly increased inclusions of alternative exons, all of which have previously been defined as neural targets of *SRRM4*.^10^ Note that the alternative exons in *AP1S2*, *ERGIC3*, and *SH3GLB1* were not part of the mRNA transcripts detected in non‐*SRRM4*‐activated fibroblast cells (sgNT). [Color figure can be viewed at wileyonlinelibrary.com]

*SRRM4*‐related AS patterns with functional roles in the brain are recapitulated in non‐neural cell types when the expression of the gene is induced.^10^ Seeking to define a set of genes in which exon inclusion was dependent on *SRRM4* in our CRISPRa‐competent fibroblast model, we performed rMATS‐turbo analysis^28^ and identified 131 significantly increased splicing‐in events in 111 genes in *SRRM4*‐activated control fibroblasts compared with their non‐*SRRM4*‐activated counterparts (FDR <0.05 and exon‐inclusion level difference >0.05) (Table S1). Of these, 46 (46/131, 35.1%) overlapped with previously characterized *SRRM4*‐regulated alternative exon‐inclusion events in neural and HEK293T cells^10^ (Fig. 3A; Table S1), highlighting the fidelity of our approach. To test the downstream functional impact of a mutant +2 residue of the splice‐donor site of *SRRM4* intron 5, we concentrated the analysis on the 111 pre‐defined genes that were affected by *SRRM4*‐dependent AS in control fibroblasts. rMATS‐turbo^28^ detected 19 significantly altered exon‐inclusion events in 13 of the 111 genes (11.7%) between control and patient 1's *SRRM4*‐activated fibroblasts (FDR <0.05 and exon‐inclusion level difference >0.05) (Table S2), consistent with a variant‐related misregulation of AS. The results were verified by direct visualizations of the rMATS‐turbo‐identified events in srRNA‐seq and/or lrRNA‐seq data from patient 1 and the controls (Fig. 3B). There was, for instance, a highly significant upregulation in the inclusion of a widely studied,^10^, ^14^ *SRRM4*‐dependent 9‐nucleotide microexon in the mRNA of *AP1S2* (FDR = 7.94 × 10^−5^; exon‐inclusion level difference = 0.16; rMATS‐turbo) (Fig. 3B; Table S2), the gene associated with X‐linked Pettigrew syndrome (OMIM: 304340).^12^, ^15^ This abnormally spliced gene represented an important candidate contributor to the *SRRM4* splicing mutation‐associated phenotype. Comparing the misregulated exon‐inclusion events to the previously described targets of SRRM4,^10^ we determined an overlap of 21.1% (4/19) (Fig. 3B; Table S2).

## Discussion

We describe three independent pediatric individuals with multisymptomatic neurodevelopmental movement disorders in whom variants at the identical +2 position of the splice‐donor site of intron 5 of *SRRM4* were identified. *SRRM4*, a crucial regulator of neural splicing programs, is highly expressed in brain regions relevant to motor control.^7^, ^21^ The proposition that the variants were causally implicated in the patients' phenotypes was supported by (1) their observed link to shared clinical outcomes with manifestations of infantile‐onset hyperkinetic syndromes in all three subjects; (2) their de novo origin; (3) their absence in the general population; and (4) the findings from functional studies focusing on transcriptomics, which revealed aberrant *SRRM4* pre‐mRNA processing as well as downstream changes in AS for one patient with available fibroblasts. The three reported patients developed movement disorders with prominent dystonia and/or chorea during their first 2 years of life, resembling the presentations of other rare monogenic conditions such as *GNAO1*‐ and *ADCY5*‐related diseases.^12^ In fact, our patients had undergone prior non‐diagnostic genetic testing with a particular focus on these entities. Additional features including developmental delay and speech impairment were shared by the individuals. As described for many other monogenic hyperkinetic movement disorders starting early in life,^32^ phenotypic heterogeneity was evident within this series of three unrelated patients, especially with regard to the predominance of certain motor manifestations determined during different examinations at varying ages of the subjects (see Videos 1 and 2). Despite the observed clinical variability, literature on published disorders linked to variants in AS‐machinery factors underscores important phenotypic commonalities^3^, ^4^, ^5^ with the here‐reported *SRRM4*‐related condition, ranging from milestone delay to epilepsy and specific movement disorders such as dystonia.^2^ Notably, another member of the SR‐related protein family, *SRRM2*, has recently been shown to cause autosomal dominant neurodevelopmental phenotypes with predominant speech dysfunction (OMIM: 620439).^12^, ^33^ Our data from srRNA‐seq and lrRNA‐seq experiments in *SRRM4*‐activated fibroblast cell lines demonstrated that mutation of the +2 position of the affected splice‐donor site had a disruptive effect on *SRRM4* pre‐mRNA splicing, creating two new transcript isoforms without frameshifts. We considered the hypothesis that the mutant isoforms, alone or in combination, could exert disease‐relevant molecular effects other than loss‐of‐function, such as change‐ or gain‐of‐function. This would be consistent with the finding that *SRRM4*‐mRNA levels were not decreased in the studied *SRRM4*‐activated patient cells compared to activated controls, as well as constraint metrics from gnomAD‐v4.1 indicating that *SRRM4* is tolerant to heterozygous loss‐of‐function changes (pLI = 0). It is also clear from information in gnomAD‐v4.1 that a nearby *SRRM4* canonical splice‐site variant (c.464+1G>A) is found in population controls. Therefore, we hypothesize that the pathogenic consequence of the patient‐specific variants may mainly involve the detected in‐frame exon‐elongation event (~35% in lrRNA‐seq data; predictably resulting in the insertion of 23 amino acids) (Fig. 2C–E), as evidenced by our observation of an abrogated nonsense codon uniquely predicted for c.464+2T>C and c.464+2T>A in the elongated sequence (Fig. 2E and Fig. S4). Further experiments are needed to decipher mRNA isoform‐related pathomechanisms in the context of the *SRRM4* splice‐site mutations. It should also be an important future objective to determine whether the elongated *SRRM4*‐mRNA isoform that we identified in association with the recurrent *SRRM4* splice‐site variant could also be found to a certain physiological extent in neuronal cells. To the best of our knowledge, there have been no previous studies reporting on the presence of the particular elongated isoform in mammalian cells. It is likely that this elongation of *SRRM4* transcripts is patient‐specific, representing a unique pathogenic event driving disease only in the context of the here‐discovered rare splice‐site variants. Nevertheless, unbiased transcriptomic assessments of different neural cell types would be helpful to better clarify the composition of *SRRM4*‐mRNA isoforms during development and in adult life. Moreover, studies based on patient‐derived neural cells will be necessary to specifically evaluate the relative expression levels of the *SRRM4* splice mutation‐associated mRNA isoforms compared to the normally spliced transcripts in the context of disease. Demonstration of changes in the inclusion levels of previously known *SRRM4*‐dependent exon targets^10^ in cells from one affected individual provided an additional line of evidence to buttress a pathogenic role for the variant. In our *SRRM4*‐expressing fibroblast model generated by genome editing, there was a misregulated inclusion of exons in genes with critical roles in brain development and function, in keeping with reported outcomes from *SRRM4* perturbation in neural cells.^10^ Our experimental approach aimed to establish high‐confidence targets of *SRRM4* that we could replicate in the CRISPRa‐treated fibroblast model, through stepwise comparisons of splicing‐in events in *SRRM4*‐activated versus non‐activated control fibroblasts and *SRRM4*‐activated control versus *SRRM4*‐activated patient cells. The analysis followed rigorous statistical‐test recommendations for such transcriptomic studies.^28^ We successfully revealed potential critical events with significant splicing deregulation in the patient fibroblasts, while we chose to focus on the most robust hits that have previously been reported to be *SRRM4*‐dependent in neural cells as well.^10^ An important question is that of the splicing‐misregulated genes are the main drivers of the phenotypes of our patients. We postulate a key candidate emerging from our experiments, *AP1S1*. In this gene, we identified an exon with highly significantly increased inclusion in the fibroblasts carrying a patient mutation. Intriguingly, the misspliced exon corresponded to an *AP1S2* neural microexon, which has been characterized as a key abnormal AS event in brain samples from subjects with autism.^14^ This microexon is required for neurotypical protein–protein interactions in the adaptor protein‐1 complex, participating in endocytic transport and ensuring the proper localization of synaptic components.^14^ Moreover, *AP1S2* is linked to a monogenic disease that features symptoms seen in our patients. Although the contribution of perturbed splicing substrates of AS regulators to disease phenotypes has been highlighted,^2^, ^3^, ^4^ more work is necessary to precisely elucidate the role for this target in the etiology of the patients' conditions. We clearly acknowledge that there may be a contribution of several different misspliced events to our patients' phenotypes. In particular, the target genes provided with this publication as Supplementary Material (please see Tables S1 and S2) may be considered as additional promising candidates for disease relevance, and we have validated the effects of the patient mutation on altered splicing for *SRRM4*'s known neural targets *ASPH*, *ERGIC3*, and *SH3GLB1* by lrRNA‐seq (see Fig. 3B). Similarly to *AP1S2*, *SH3GLB1* is known to be involved in the endosomal‐autophagosomal‐lysosomal pathway,^34^ suggesting potential convergence on established dystonia‐related mechanisms.^35^ Alternatively, the full composition of disease‐relevant targets may not be captured in our present CRISPRa‐based fibroblast model given that many *SRRM4*‐dependent splicing events are essentially neural‐specific. Recent work has shown that *SRRM4* expression levels impact sensitively on complex AS networks by influencing specific 3′ splice‐site architectures of microexons, mostly in a developmental window‐ and cell type‐specific manner.^36^ Therefore, any changes in the availability and/or integrity of *SRRM4*‐mRNA transcripts may perturb finer regulations of microexon landscapes required for the development of dystonia/chorea‐associated brain regions (Fig. S6). A crucial step forward will be to comprehensively evaluate the wider spectrum of missplicing events in patient‐derived neural cells. The utilization of CRISPRa‐competent fibroblasts as a sub‐optimal model to monitor downstream AS events related to *SRRM4* is a key limitation of this study. Hence, we were unable to recapitulate previously reported *SRRM4*‐controlled events with potential relevance to the clinical features of our patients, such as AS of a microexon in the dystonia‐linked gene *TAF1*.^11^ Furthermore, although high gene activation can generally be achieved via CRISPRa, efficiency varies between genes, depending on chromatin state and the specific activation system.^22^, ^23^

In conclusion, our work presents compelling evidence for specific *SRRM4* splice‐site variants as a novel cause of an infantile‐onset mixed hyperkinetic disorder with neurodevelopmental impairment. Misregulation of specific AS events may lead to the observed phenotypic abnormalities, offering opportunities for future therapeutic modulation of this unique form of genetic dystonia and chorea.

## Author Roles

(1) Research Project: A. Conception, B. Organization, C. Execution; (2) Statistical Analysis: A. Design, B. Execution, C. Review and Critique; (3) Manuscript Preparation: A. Writing of the First Draft, B. Review and Critique.

P.H.: 1A, 1C, 2A, 2B, 2C, 3A

V.K.: 1A, 1C, 3B

A.S.: 1A, 1C, 2A, 2B, 3B

A.K.: 1C, 3B

S.Z.: 1C, 3B

R.S.: 1C, 3B

F.K.: 1C, 3B

M.B.: 1C, 3B

S.K.: 1C, 3B

U.S.: 1C, 3B

C.Z.: 1C, 3B

I.D.: 1C, 3B

M.P.: 1C, 3B

E.G.: 1C, 3B

A.M.S.: 1C, 3B

P.M.K.: 1C, 3B

C.W.: 1C, 3B

S.B.: 1C, 3B

F.A.: 1C, 3B

S.B.: 1C, 3B

J.W.: 1B, 1C, 3B

K.O.: 1B, 1C, 2C, 3B

I.K.: 1B, 1C, 3B

G.C.K.: 1B, 1C, 3B

M.Z.: 1A, 1B, 1C, 2A, 2C, 3A

## Financial Disclosures and Conflicts of Interest

The authors report no disclosures. Author disclosures are available in the Supporting Information.

## Supporting information


**Figure S1.** cDNA analysis of *SRRM4* expression following CRISPR activation (CRISPRa).
**Figure S2.** *SRRM4* pre‐mRNA splicing consequences associated with patient 1's recurrent c.464+2T>C variant across all biological replicates.
**Figure S3.** Short‐read RNA sequencing (srRNA‐seq) data‐based quantification of *SRRM4* expression following CRISPR activation (CRISPRa).
**Figure S4.** A premature termination codon (PTC)‐encoding TAA triplet in *SRRM4* intron 5 is not affected by variants in gnomAD v4.1.
**Figure S5.** Predicted protein consequences associated with patient 1's recurrent *SRRM4* c.464+2T>C variant.
**Figure S6.** Schematic depiction of proposed SRRM4 variant‐associated disease pathogenesis.
**Table S1.** List of 131 significant splicing‐in events (FDR0.05) in 111 genes in *SRRM4*‐activated control fibroblasts compared to their non‐*SRRM4*‐activated counterparts (rMATS‐turbo analysis1).
**Table S2.** List of 19 significantly altered exon‐inclusion events (FDR0.05) in 13 of the 111 pre‐defined genes in *SRRM4*‐activated patient fibroblasts compared to *SRRM4*‐activated control fibroblasts (rMATS‐turbo analysis^1^).

## Data Availability

The data that support the findings of this study are available on request from the corresponding author. The data are not publicly available due to privacy or ethical restrictions.
