# Supplementary material for: Neurodevelopmental Disorder with Dystonia and Chorea Linked to De Novo Variants in the Splicing Regulator SRRM4
Source: Mov Disord. 2026 Apr 9;41(7):1704–15. doi: 10.1002/mds.70297 (PMC13387927; doi:10.1002/mds.70297)
Supplement: Supplementary file 3 — Figure S1. cDNA analysis of SRRM4 expression following CRISPR activation (CRISPRa). Figure S2. SRRM4 pre‐mRNA splicing consequences associated with patient 1's recurrent c.464+2T>C variant across all biological replicates. Figure S3. Short‐read RNA sequencing (srRNA‐seq) data‐based quantification of SRRM4 expression following CRISPR activation (CRISPRa). Figure S4. A premature termination codon (PTC)‐encoding TAA triplet in SRRM4 intron 5 is not affected by variants in gnomAD v4.1. Figure S5. Predicted protein consequences associated with patient 1's recurrent SRRM4 c.464+2T>C variant. Figure S6. Schematic depiction of proposed SRRM4 variant‐associated disease pathogenesis. Table S1. List of 131 significant splicing‐in events (FDR0.05) in 111 genes in SRRM4‐activated control fibroblasts compared to their non‐SRRM4‐activated counterparts (rMATS‐turbo analysis1). Table S2. List of 19 significantly altered exon‐inclusion events (FDR0.05) in 13 of the 111 pre‐defined genes in SRRM4‐activated patient fibroblasts compared to SRRM4‐activated control fibroblasts (rMATS‐turbo analysis 1 ). [file MDS-41-1704-s001.pdf]

SUPPLEMENTARY MATERIAL

Table of contents

Supplementary Methods

Supplementary Figures

Figure S1 cDNA analysis of *SRRM4* expression following CRISPR activation (CRISPRa)

Figure S2 *SRRM4* pre-mRNA splicing consequences associated with patient 1`s recurrent c.464+2T>C variant across all biological replicates

Figure S3 Short-read RNA sequencing (srRNA-seq) data-based quantification of *SRRM4* expression following CRISPR activation (CRISPRa)

Figure S4 A premature termination codon (PTC)-encoding TAA triplet in *SRRM4* intron 5 is not affected by variants in gnomAD v4.1

Figure S5 Predicted protein consequences associated with patient 1`s recurrent *SRRM4* c.464+2T>C variant

Figure S6 Schematic depiction of proposed *SRRM4* variant-associated disease pathogenesis

Supplementary Tables

Table S1 List of 131 significant splicing-in events (FDR<0.05 and ΔPSI >0.05) in 111 genes in *SRRM4*-activated control fibroblasts compared to their non-*SRRM4*-activated counterparts (rMATS-turbo analysis[1])

Table S2 List of 19 significantly altered exon-inclusion events (FDR<0.05 and ΔPSI >0.05) in 13 of the 111 pre-defined genes in *SRRM4*-activated patient fibroblasts compared to *SRRM4*-activated control fibroblasts (rMATS-turbo analysis[1])

Supplementary References

Supplementary Methods

Fibroblast culture and induced SRRM4 expression

Fibroblast lines established from skin-biopsy specimens were cultured in DMEM (Gibco #41966052) supplemented with 10% FBS, Penicillin-Streptomycin (Gibco #15140122) and 0.2 mM uridine at 37°C, 5% CO_2 in a humidified atmosphere incubator, as described[2]. To activate *SRRM4* expression in fibroblasts, we used a lentiviral CRISPR-activation (CRISPRa) system[3], expressing a specific guide RNA (sg*SRRM4*, 5'-GAGGGATCAGCGCTGCAACT-3') directed to the promoter region of the canonical isoform of *SRRM4* (NM_194286.4). A non-targeting guide (sgNT, 5'-ACGGAGGCTAAGCGTCGCAA-3') was used as control. The CRISPRa system was comprised of three transfer vectors, namely lenti sgRNA(MS2)_puro optimized backbone, lenti dCAS-VP64_Blast, and lentiMPHv2 (Addgene plasmids # 73797, # 61425 and # 89308, gifts from Feng Zhang). Lentivirus was generated as previously described[4], using envelope plasmids pCAG_Eco (Addgene Plasmid #35617, gift from Arthur Nienhuis & Patrick Salmo) or pMD2.G, together with psPAX2 packaging plasmid (Addgene Plasmids #12259 and #12260, gifts from Didier Trono). For sgRNA(MS2)_puro optimized backbone we generated ecotropic murine lentivirus using pCAG_Eco. Fibroblasts were seeded at 5,000 cells per well in a 24 well plate 24h prior to transduction. Cells were then co-transduced with three lentiviruses using the 5 µl of Ecotropic Receptor Booster (Takara # 631471) per well, and treated according to the manufacturer's protocol. 48h after infection, 75 µg/ml hygromycin, 5 µg/ml blasticidine and 1 µg/ml puromycin were added to the culture media for 7 days, to select for triple-infected cells. Cells were passaged 3 times, expanded until 90% confluency on a 10 cm dish was reached and harvested for RNA extraction.

Verification of SRRM4 expression

Isolated RNA was reverse-transcribed using the High-Capacity cDNA Reverse Transcription Kit (Applied Biosystems). Expression of *SRRM4* was then assessed by PCR using primers spanning exons 2-7 (*SRRM4*-F: 5'-CAGAGCCCCAGAATAACCCC-3', *SRRM4*-R: 5'-GCTGACAGGTAGCCAAGCAT-3'), followed by agarose gel electrophoresis

Supplementary Figures

Suppl. Figure 1 cDNA analysis of *SRRM4* expression following CRISPR activation (CRISPRa)

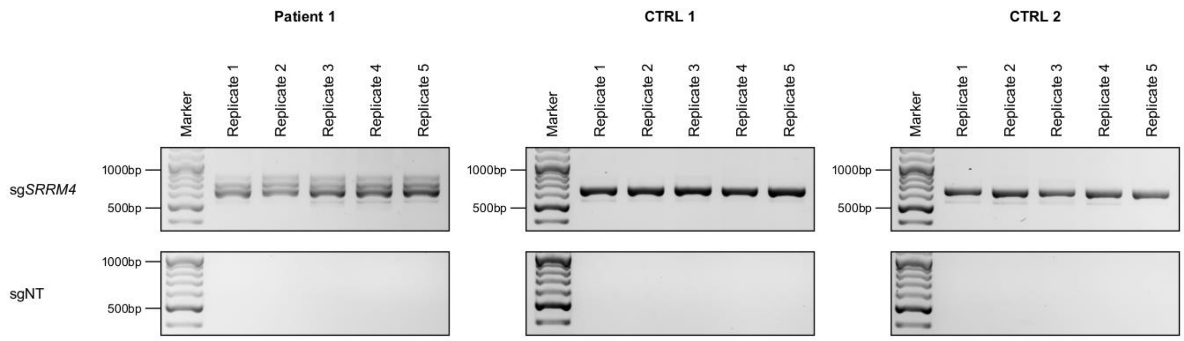

Agarose gels showing *SRRM4* RT-PCR amplicons produced from cDNA of *SRRM4*-activated fibroblast cell lines (sg*SRRM4*) from patient 1 and the control subjects (CTRL 1, CTRL 2). Five biological replicates were analyzed for each subject (patient 1, CTRL 1, CTRL 2). RT-PCT primers were designed to span *SRRM4* exons 2-7. Note the presence of three different RT-PCR products for patient 1. No RT-PCR amplicons were detected in non-*SRRM4*-activated fibroblast cell lines (sgNT) from the three subjects.

Suppl. Figure 2 *SRRM4* pre-mRNA splicing consequences associated with patient 1`s recurrent c.464+2T>C variant across all biological replicates

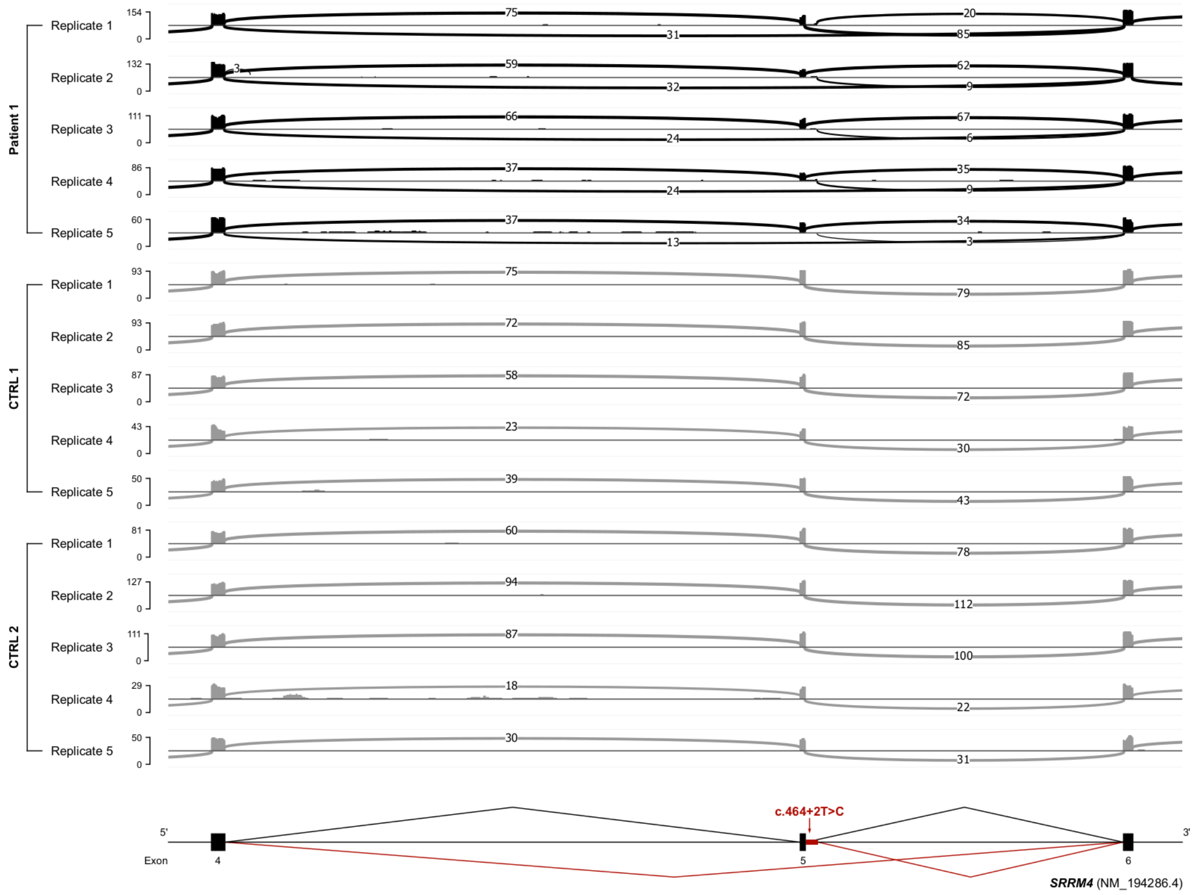

Sashimi plots established from srRNA-seq data generated for *SRRM4*-activated fibroblast cell lines from patient 1 and the control subjects (CTRL 1, CTRL 2) are shown. Five biological replicates were analyzed for each subject (patient 1, CTRL 1, CTRL 2). CRISPRa, CRISPR activation; srRNA-seq, short-read RNA sequencing.

Suppl. Figure 3 Short-read RNA sequencing (srRNA-seq) data-based quantification of *SRRM4* expression following CRISPR activation (CRISPRa)

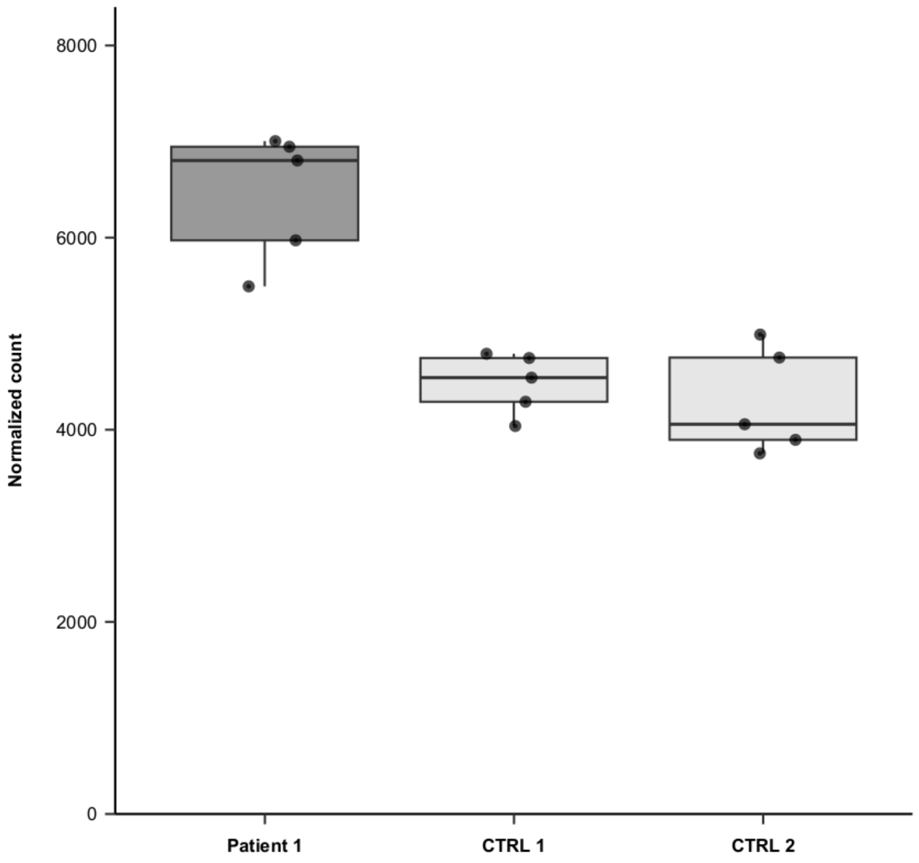

Box plots showing the results from quantitative analysis of *SRRM4* expression in *SRRM4*-activated fibroblast cell lines from patient 1 and the control subjects (CTRL 1, CTRL 2). For the quantifications, srRNA-seq data were analyzed using DESeq2[5]. Five biological replicates were included in the analysis for each subject (patient 1, CTRL 1, CTRL 2).

Suppl. Figure 4 A premature termination codon (PTC)-encoding TAA triplet in *SRRM4* intron 5 is not affected by variants in gnomAD v4.1

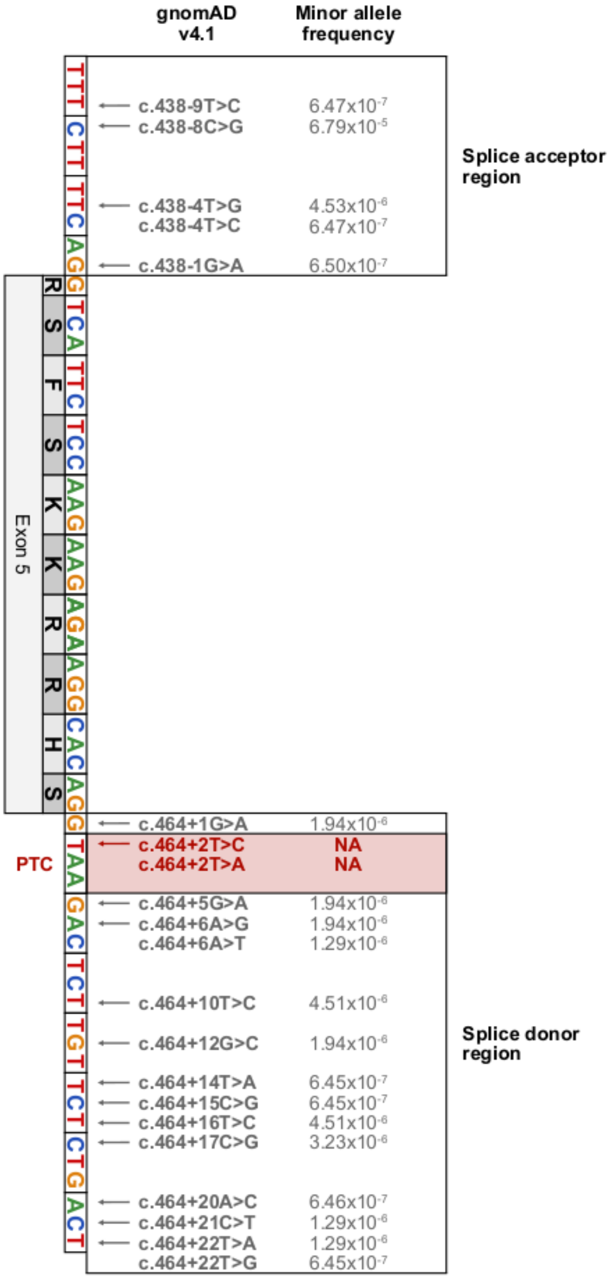

The patient-specific c.464+2T>C and c.464+2T>A variants were predicted to affect the invariant PTC. Note gnomAD v4.1 variants at positions +1, +5, and +6 but not at the PTC-encoding positions +2, +3, and +4. NA, not available.

Suppl. Figure 5 Predicted protein consequences associated with patient 1`s recurrent *SRRM4* c.464+2T>C variant

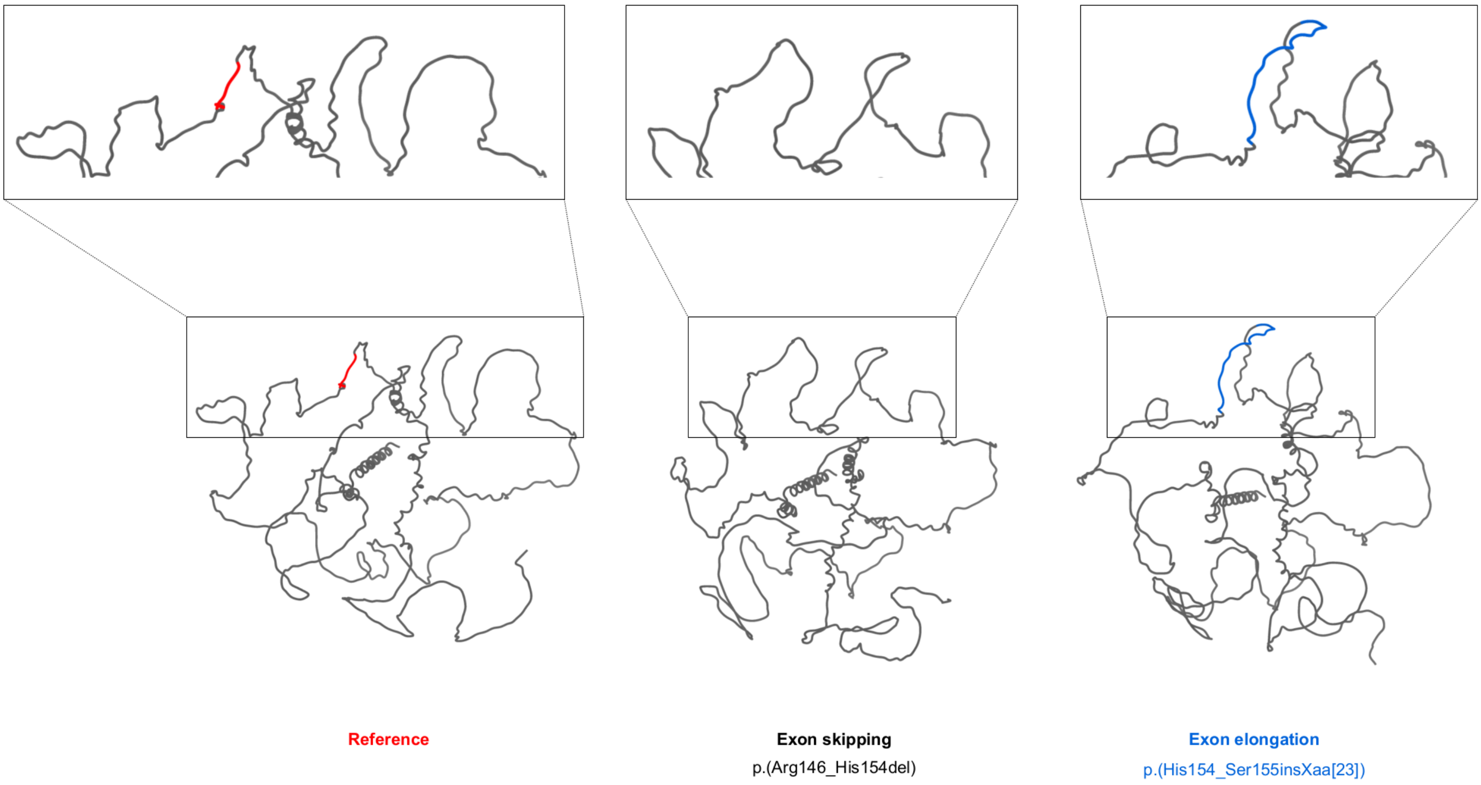

Structural effects on a 3D model of SRRM4 predicted by AlphaFold2[6] and visualized by UCSF ChimeraX[7] are illustrated for the predicted protein changes resulting from the mutation of the splice-donor site of intron 5 of *SRRM4*. The affected protein regions are highlighted in the zoom-in panels, with annotations of the predicted protein effects as follows (annotations based on NP_919262.2): exon skipping, p.(Arg146_His154del); exon elongation, p.(His154_Ser155insXaa[23]).

Suppl. Figure 6 Schematic depiction of proposed *SRRM4* variant-associated disease pathogenesis

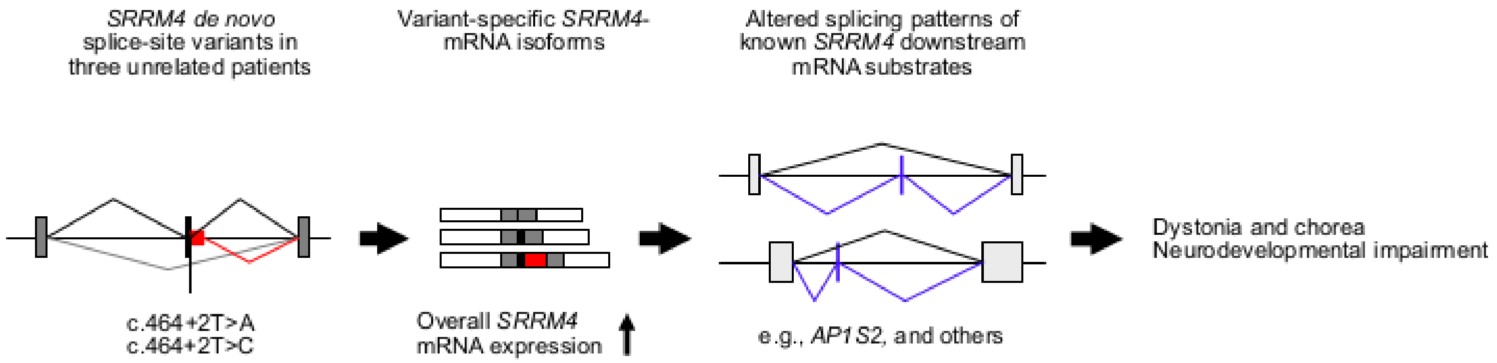

The altered *SRRM4* splice site is associated with new *SRRM4* mRNA-isoforms and increased overall *SRRM4* mRNA levels, consistent with a potenital toxic gain-of-function effect, and resulting downstream perturbation of alternative splicing control.

Supplementary Tables

Suppl. Table 1 List of 131 significant splicing-in events (FDR<0.05 and ΔPSI >0.05) in 111 genes in *SRRM4*-activated control fibroblasts compared to their non-*SRRM4*-activated counterparts (rMATS-turbo analysis[1])

Gene ID	Gene symbol	Chromosome	Exon start	Exon end	P value	FDR	Inclusion level difference	Raj et al.[8] N2A [gene]	Raj et al.[8] N2A [event]
ENSG00000116337.15_5	*AMPD2*	chr1	110167924	110168409	6.63E-05	0.016444242	0.26	No	No
ENSG00000118200.14_3	*CAMSAP2*	chr1	200797700	200797733	4.14E-06	0.001671941	0.088	No	No
ENSG00000116406.18_3	*EDEM3*	chr1	184670630	184670678	8.32E-06	0.002950034	0.111	No	No
ENSG00000116406.18_3	*EDEM3*	chr1	184670663	184670678	2.05E-04	0.039612673	0.088	No	No
ENSG00000155816.19_3	*FMN2*	chr1	240343468	240343480	5.11E-15	3.00E-11	0.134	No	No
ENSG00000174842.16_2	*GLMN*	chr1	92732256	92732298	1.93E-04	0.038061979	0.157	No	No
ENSG00000054523.17_4	*KIF1B*	chr1	10333071	10333089	1.56E-05	0.004967893	0.12	Yes	Yes
ENSG00000163875.15_3	*MEAF6*	chr1	37962307	37962337	3.46E-07	2.24E-04	0.215	Yes	Yes
ENSG00000116604.17_4	*MEF2D*	chr1	156446285	156446306	1.01E-11	3.07E-08	0.192	Yes	Yes
ENSG00000097033.14_3	*SH3GLB1*	chr1	87194085	87194172	2.12E-11	5.89E-08	0.053	Yes	Partial
ENSG00000097033.14_3	*SH3GLB1*	chr1	87194085	87194124	7.88E-10	1.37E-06	0.054	Yes	Yes
ENSG00000097033.14_3	*SH3GLB1*	chr1	87195770	87195794	1.11E-16	8.70E-13	0.083	Yes	Yes
ENSG00000115657.13_4	*ABCB6*	chr2	220082391	220082529	7.49E-05	0.018190743	0.225	No	No
ENSG00000143933.16_3	*CALM2*	chr2	47399552	47399598	2.20E-05	0.006669344	0.192	No	No
ENSG00000172292.14_3	*CERS6*	chr2	169622831	169622855	1.15E-06	5.99E-04	0.144	No	No
ENSG00000204843.12_3	*DCTN1*	chr2	74590740	74590755	0	0	0.159	Yes	Yes
ENSG00000144036.15_3	*EXOC6B*	chr2	72410022	72410034	7.22E-05	0.017668463	0.102	No	No
ENSG00000115414.18_4	*FN1*	chr2	216236631	216237045	1.50E-05	0.004791491	0.237	No	No
ENSG00000115419.12_4	*GLS*	chr2	191777918	191778090	5.18E-05	0.013524972	0.141	No	No
ENSG00000144366.15_6	*GULP1*	chr2	189454466	189454477	2.79E-04	0.047487563	0.183	No	No
ENSG00000151690.14_3	*MFSD6*	chr2	191364557	191364582	7.57E-06	0.002757969	0.154	Yes	Yes
ENSG00000061676.14_3	*NCKAP1*	chr2	183889705	183889723	0	0	0.248	No	No
ENSG00000189223.14_3	*PAX8-AS1*	chr2	114012966	114013126	2.98E-06	0.001303145	0.338	No	No

ENSG00000189223.14_3	*PAX8-AS1*	chr2	114016840	114017161	7.74E-06	0.002797177	0.222	No	No
ENSG00000055917.15_4	*PUM2*	chr2	20457997	20458131	3.79E-06	0.001569435	0.416	No	No
ENSG00000115694.14_3	*STK25*	chr2	242447420	242447550	1.09E-04	0.024363291	0.248	No	No
ENSG00000060971.17_3	*ACAA1*	chr3	38167652	38168115	8.21E-05	0.019641849	0.059	No	No
ENSG00000060971.17_3	*ACAA1*	chr3	38168000	38168115	9.79E-07	5.38E-04	0.373	No	No
ENSG00000060971.17_3	*ACAA1*	chr3	38168000	38168186	1.12E-06	5.93E-04	0.373	No	No
ENSG00000060971.17_3	*ACAA1*	chr3	38168000	38168200	1.12E-06	5.93E-04	0.373	No	No
ENSG00000114331.13_4	*ACAP2*	chr3	195046172	195046184	1.07E-05	0.003699884	0.12	Yes	Yes
ENSG00000161203.13_3	*AP2M1*	chr3	183898432	183898438	2.01E-04	0.039200009	0.102	No	No
ENSG00000163539.16_3	*CLASP2*	chr3	33615964	33615988	2.22E-05	0.006706592	0.16	Yes	Yes
ENSG00000114735.9_3	*HEMK1*	chr3	50609507	50609639	2.44E-04	0.044080821	0.167	No	No
ENSG00000114670.13_2	*NEK11*	chr3	130887673	130887781	1.12E-05	0.00379335	0.225	No	No
ENSG00000174748.20_5	*RPL15*	chr3	23959023	23959340	8.01E-05	0.019201791	0.151	No	No
ENSG00000174748.20_5	*RPL15*	chr3	23959327	23959340	1.70E-04	0.034569245	0.226	No	No
ENSG00000163697.16_4	*APBB2*	chr4	40859042	40859048	4.89E-05	0.012966296	0.123	No	No
ENSG00000181982.18_2	*CCDC149*	chr4	24822610	24822709	4.56E-05	0.012282297	0.08	No	No
ENSG00000181982.18_2	*CCDC149*	chr4	24822676	24822709	1.00E-08	1.05E-05	0.171	No	No
ENSG00000075539.14_4	*FRYL*	chr4	48504844	48504862	8.22E-06	0.002936096	0.164	Yes	Yes
ENSG00000084093.16_2	*REST*	chr4	57793780	57793830	3.51E-06	0.001487736	0.141	No	No
ENSG00000151612.15_4	*ZNF827*	chr4	146684241	146684274	5.80E-07	3.44E-04	0.517	No	No
ENSG00000113108.19_3	*APBB3*	chr5	139941428	139941434	1.37E-07	1.02E-04	0.402	No	No
ENSG00000081189.15_5	*MEF2C*	chr5	88026027	88026051	4.87E-06	0.001915515	0.227	No	No
ENSG00000120708.16_3	*TGFBI*	chr5	135389631	135390105	1.72E-04	0.034697329	0.093	No	No
ENSG00000047932.13_4	*GOPC*	chr6	117898610	117898634	5.18E-10	9.95E-07	0.254	Yes	Yes
ENSG00000135318.11_3	*NT5E*	chr6	86201694	86201816	9.03E-06	0.003179608	0.358	No	No
ENSG00000152894.14_5	*PTPRK*	chr6	128322340	128322352	2.88E-07	1.91E-04	0.104	No	No
ENSG00000152894.14_5	*PTPRK*	chr6	128324341	128324377	4.84E-06	0.001910863	0.09	No	No
ENSG00000198818.9_2	*SFT2D1*	chr6	166744872	166744895	1.09E-07	8.26E-05	0.131	No	No
ENSG00000111850.10_3	*SMIM8*	chr6	88040409	88040430	2.42E-04	0.044080821	0.187	No	No
ENSG00000285953.1_1	*AC000120.3*	chr7	91874215	91874448	2.15E-04	0.040538607	0.483	No	No

ENSG00000075624.14_4	*ACTB*	chr7	5567363	5567554	2.97E-04	0.049707554	0.544	No	No
ENSG00000133612.18_2	*AGAP3*	chr7	150835229	150835400	7.72E-05	0.018654616	0.192	No	No
ENSG00000153956.15_3	*CACNA2D1*	chr7	81612629	81612650	2.31E-07	1.60E-04	0.155	No	No
ENSG00000106554.12_4	*CHCHD3*	chr7	132571733	132571748	0	0	0.196	No	No
ENSG00000049540.16_4	*ELN*	chr7	73480273	73480327	1.36E-05	0.004407993	0.225	No	No
ENSG00000006652.13_4	*IFRD1*	chr7	112102324	112102433	6.39E-05	0.016070293	0.139	No	No
ENSG00000091127.13_2	*PUS7*	chr7	105137400	105137418	2.69E-05	0.007976862	0.134	Yes	Yes
ENSG00000127990.17_4	*SGCE*	chr7	94217089	94217124	0	0	0.1	Yes	Yes
ENSG00000153317.14_3	*ASAP1*	chr8	131173030	131173039	7.07E-13	3.16E-09	0.121	Yes	Yes
ENSG00000198363.17_3	*ASPH*	chr8	62594997	62595042	0	0	0.183	Yes	Yes
ENSG00000169398.19_4	*PTK2*	chr8	141772466	141772487	7.87E-13	3.36E-09	0.164	Yes	Yes
ENSG00000078668.13_3	*VDAC3*	chr8	42254195	42254198	3.01E-11	7.86E-08	0.135	No	No
ENSG00000165802.22_5	*NSMF*	chr9	140350080	140350086	8.80E-08	6.84E-05	0.109	No	No
ENSG00000119396.10_3	*RAB14*	chr9	123952831	123953008	1.75E-04	0.035128782	0.051	No	No
ENSG00000148341.17_3	*SH3GLB2*	chr9	131771731	131771746	4.03E-14	2.23E-10	0.113	Yes	Yes
ENSG00000197694.15_5	*SPTAN1*	chr9	131371929	131371944	0	0	0.161	Yes	Yes
ENSG00000197579.7_2	*TOPORS*	chr9	32550771	32550966	9.54E-05	0.021926665	0.236	No	No
ENSG00000160293.16_4	*VAV2*	chr9	136652367	136652382	8.82E-05	0.02087501	0.12	Yes	Yes
ENSG00000160293.16_4	*VAV2*	chr9	136675312	136675327	3.41E-05	0.009773681	0.105	Yes	Yes
ENSG00000107897.18_3	*ACBD5*	chr10	27512135	27512168	9.14E-05	0.021477637	0.191	Yes	Yes
ENSG00000107863.17_4	*ARHGAP21*	chr10	24911661	24911691	4.20E-11	1.07E-07	0.132	No	No
ENSG00000107863.17_4	*ARHGAP21*	chr10	24911661	24911804	4.30E-07	2.64E-04	0.056	No	No
ENSG00000148634.15_3	*HERC4*	chr10	69718869	69718893	2.01E-13	1.05E-09	0.138	Yes	Yes
ENSG00000096746.17_2	*HNRNPH3*	chr10	70098259	70098444	4.72E-05	0.012572793	0.066	No	No
ENSG00000197746.13_3	*PSAP*	chr10	73583644	73583650	5.55E-16	4.01E-12	0.125	Yes	Partial
ENSG00000197746.13_3	*PSAP*	chr10	73583644	73583653	2.19E-04	0.041019046	0.205	Yes	Yes
ENSG00000151532.13_2	*VTI1A*	chr10	114293288	114293309	3.75E-08	3.23E-05	0.209	Yes	Yes
ENSG00000166313.18_5	*APBB1*	chr11	6423206	6423212	1.58E-11	4.63E-08	0.092	Yes	Yes
ENSG00000167996.15_3	*FTH1*	chr11	61732869	61733001	2.13E-04	0.040477528	0.523	No	No
ENSG00000110514.19_3	*MADD*	chr11	47330530	47330593	1.27E-04	0.027592416	0.054	Yes	Yes

ENSG00000131626.18_5	*PPFIA1*	chr11	70197099	70197129	2.11E-05	0.006471834	0.501	Yes	Yes
ENSG00000131626.18_5	*PPFIA1*	chr11	70197099	70197129	3.19E-05	0.009273057	0.091	Yes	Yes
ENSG00000154134.14_2	*ROBO3*	chr11	124747861	124748332	2.71E-04	0.046438405	0.276	No	No
ENSG00000154134.14_2	*ROBO3*	chr11	124748193	124748332	2.45E-04	0.044080821	0.27	No	No
ENSG00000102189.16_2	*EEA1*	chr12	93240037	93240052	2.79E-04	0.047487563	0.066	Yes	Yes
ENSG00000139436.20_4	*GIT2*	chr12	110405118	110405124	1.09E-08	1.12E-05	0.159	Yes	Yes
ENSG00000051825.14_5	*MPHOSPH9*	chr12	123712009	123712163	5.67E-06	0.002175976	0.361	No	No
ENSG00000136021.18_3	*SCYL2*	chr12	100711458	100711470	4.90E-05	0.012966296	0.074	No	No
ENSG00000182796.14_4	*TMEM198B*	chr12	56225040	56225133	1.38E-04	0.029561537	0.244	No	No
ENSG00000180776.15_5	*ZDHHC20*	chr13	21967097	21967133	1.33E-06	6.74E-04	0.122	Yes	Yes
ENSG00000198513.11_3	*ATL1*	chr14	51096712	51096727	5.97E-10	1.08E-06	0.371	No	No
ENSG00000125375.14_4	*ATP5S*	chr14	50790662	50790878	1.54E-04	0.032180049	0.238	No	No
ENSG00000125375.14_4	*ATP5S*	chr14	50790662	50790834	1.60E-04	0.033219987	0.145	No	No
ENSG00000182979.17_5	*MTA1*	chr14	105934674	105934686	4.49E-08	3.77E-05	0.152	Yes	Yes
ENSG00000182718.16_5	*ANXA2*	chr15	60686772	60686894	1.00E-04	0.022942745	0.379	No	No
ENSG00000103888.16_2	*CEMIP*	chr15	81165878	81166037	3.10E-06	0.001342627	0.071	No	No
ENSG00000068305.17_4	*MEF2A*	chr15	100243566	100243590	2.28E-06	0.001082255	0.072	Yes	Yes
ENSG00000067141.16_2	*NEO1*	chr15	73567032	73567065	1.83E-07	1.32E-04	0.175	Yes	Yes
ENSG00000137817.16_3	*PARP6*	chr15	72541585	72541655	7.77E-16	5.22E-12	0.363	Yes	Yes
ENSG00000168096.14_3	*ANKS3*	chr16	4776659	4776781	1.55E-04	0.032356377	0.175	No	No
ENSG00000168096.14_3	*ANKS3*	chr16	4776979	4777178	1.18E-05	0.003957579	0.334	No	No
ENSG00000124074.11_2	*ENKD1*	chr16	67698898	67699071	2.05E-04	0.039612673	0.2	No	No
ENSG00000135709.12_3	*KIAA0513*	chr16	85116169	85116199	2.09E-04	0.040065546	0.13	Yes	Yes
ENSG00000008710.19_3	*PKD1*	chr16	2163041	2163066	1.46E-05	0.004687936	0.112	Yes	Partial
ENSG00000008710.19_3	*PKD1*	chr16	2163041	2163060	2.31E-04	0.042638163	0.093	Yes	Yes
ENSG00000140682.18_4	*TGFB1I1*	chr16	31485002	31485055	2.10E-04	0.040178146	0.074	No	No
ENSG00000140682.18_4	*TGFB1I1*	chr16	31485155	31485298	1.59E-04	0.032934606	0.055	No	No
ENSG00000033627.16_4	*ATP6V0A1*	chr17	40660589	40660607	0	0	0.308	Yes	Yes
ENSG00000108509.20_4	*CAMTA2*	chr17	4872794	4872815	2.52E-13	1.25E-09	0.209	Yes	Yes
ENSG00000108262.15_4	*GIT1*	chr17	27905979	27906006	2.43E-04	0.044080821	0.112	Yes	Yes

ENSG00000161647.18_3	*MPP3*	chr17	41894044	41894065	4.90E-12	1.59E-08	0.599	Yes	Yes
ENSG00000108523.15_3	*RNF167*	chr17	4843781	4844021	2.31E-04	0.042638163	0.345	No	No
ENSG00000008294.20_3	*SPAG9*	chr17	49085197	49085212	5.81E-07	3.44E-04	0.07	No	No
ENSG00000187824.8_5	*TMEM220*	chr17	10632347	10632377	2.11E-04	0.040232531	0.276	No	No
ENSG00000101639.18_4	*CEP192*	chr18	13038368	13038578	6.32E-05	0.015974124	0.397	No	No
ENSG00000168502.17_3	*MTCL1*	chr18	8779030	8779108	2.30E-04	0.042620191	0.058	No	No
ENSG00000089351.14_4	*GRAMD1A*	chr19	35513803	35513815	4.24E-13	1.99E-09	0.142	No	No
ENSG00000008441.16_5	*NFIX*	chr19	13189426	13189549	2.97E-08	2.58E-05	0.076	No	No
ENSG00000125991.19_4	*ERGIC3*	chr20	34142142	34142157	0	0	0.184	Yes	Yes
ENSG00000125772.12_3	*GPCPD1*	chr20	5566839	5566915	1.81E-05	0.005618177	0.223	No	No
ENSG00000132793.11_2	*LPIN3*	chr20	39986239	39986674	1.01E-04	0.022977137	0.239	No	No
ENSG00000132793.11_2	*LPIN3*	chr20	39986276	39986674	1.11E-04	0.024579465	0.24	No	No
ENSG00000124104.18_2	*SNX21*	chr20	44469086	44469097	2.00E-10	4.17E-07	0.401	Yes	Yes
ENSG00000101150.17_4	*TPD52L2*	chr20	62518916	62518958	0	0	0.064	Yes	Yes
ENSG00000100099.20_4	*HPS4*	chr22	26867178	26867256	1.52E-04	0.031990412	0.095	No	No
ENSG00000182287.14_5	*AP1S2*	chrX	15846314	15846323	0	0	0.151	Yes	Yes
ENSG00000147251.15_4	*DOCK11*	chrX	117819482	117819500	3.54E-07	2.27E-04	0.163	Yes	Yes
ENSG00000147121.15_3	*KRBOX4*	chrX	46331459	46331578	6.34E-05	0.015974124	0.17	No	No

Suppl. Table 2 List of 19 significantly altered exon-inclusion events (FDR<0.05 and ΔPSI >0.05) in 13 of the 111 pre-defined genes in *SRRM4*-activated patient fibroblasts compared to *SRRM4*-activated control fibroblasts (rMATS-turbo analysis[1])

Gene ID	Gene symbol	Chromosome	Exon start	Exon end	P value	FDR	Inclusion level difference	Category	Raj et al.[8] N2A [gene]	Raj et al.[8] N2A [event]	OMIM [MIM number]
**ENSG00000097033.14_3**	***SH3GLB1***	**chr1**	**87195770**	**87195794**	**0.001059475**	**0.032718017**	**0.092**	**High in case**	**Yes**	**Yes**	
ENSG00000189223.14_3	*PAX8-AS1*	chr2	114016840	114017161	2.80E-05	0.002038917	0.084	High in case	No	No	
ENSG00000055917.15_4	*PUM2*	chr2	20478343	20478580	0.001159442	0.034631215	0.192	High in case	No	No	
ENSG00000163697.16_4	*APBB2*	chr4	40937093	40937156	9.55E-05	0.004843029	0.081	High in case	No	No	
ENSG00000049540.16_4	*ELN*	chr7	73457447	73457498	2.80E-08	1.70E-05	0.125	High in case	No	No	123700; 185500
ENSG00000049540.16_4	*ELN*	chr7	73480023	73480062	1.24E-04	0.005941804	0.101	High in case	No	No	123700; 185500
**ENSG00000198363.17_3**	***ASPH***	**chr8**	**62594997**	**62595042**	**3.33E-05**	**0.002334018**	**0.1**	**High in case**	**Yes**	**Yes**	**601552**
ENSG00000165802.22_5	*NSMF*	chr9	140349690	140349759	6.78E-05	0.003743249	0.16	High in case	No	No	614838
ENSG00000110514.19_3	*MADD*	chr11	47295377	47295527	0.001631624	0.046450284	0.082	High in case	Yes	No	619004; 619005
ENSG00000182796.14_4	*TMEM198B*	chr12	56228634	56229399	0.001679533	0.047078596	0.077	High in case	No	No	
ENSG00000182796.14_4	*TMEM198B*	chr12	56228634	56228823	4.58E-04	0.016701949	0.092	High in case	No	No	
ENSG00000182718.16_5	*ANXA2*	chr15	60656627	60656722	1.41E-06	2.34E-04	0.132	High in case	No	No	
ENSG00000182718.16_5	*ANXA2*	chr15	60678226	60678285	1.86E-04	0.007889587	0.081	High in case	No	No	
**ENSG00000125991.19_4**	***ERGIC3***	**chr20**	**34142142**	**34142157**	**7.75E-06**	**9.20E-04**	**0.098**	**High in case**	**Yes**	**Yes**	
**ENSG00000182287.14_5**	***AP1S2***	**chrX**	**15846314**	**15846323**	**2.18E-07**	**7.94E-05**	**0.16**	**High in case**	**Yes**	**Yes**	**304340**
ENSG00000115414.18_4	*FN1*	chr2	216236631	216237047	9.57E-05	0.004843029	-0.059	High in CTRL	No	No	601894; 184255
ENSG00000115414.18_4	*FN1*	chr2	216257653	216257926	9.63E-08	4.39E-05	-0.142	High in CTRL	No	No	601894; 184255
ENSG00000182718.16_5	*ANXA2*	chr15	60678226	60678285	2.04E-04	0.008265056	-0.075	High in CTRL	No	No	
ENSG00000182718.16_5	*ANXA2*	chr15	60678231	60678285	2.03E-04	0.008265056	-0.081	High in CTRL	No	No	

Supplementary References

1. Wang Y, Xie Z, Kutschera E, Adams JI, Kadash-Edmondson KE, Xing Y. rMATS-turbo: an efficient and flexible computational tool for alternative splicing analysis of large-scale RNA-seq data. Nat Protoc 2024;19(4):1083-1104.
2. Zech M, Dzinovic I, Skorvanek M, et al. Combined genomics and proteomics unveils elusive variants and vast aetiologic heterogeneity in dystonia. Brain 2025;148(8):2827-2846.
3. Konermann S, Brigham MD, Trevino AE, et al. Genome-scale transcriptional activation by an engineered CRISPR-Cas9 complex. Nature 2015;517(7536):583-588.
4. Kittke V, Zhao C, Lam DD, et al. RLS-associated MEIS transcription factors control distinct processes in human neural stem cells. Sci Rep 2024;14(1):28986.
5. Love MI, Huber W, Anders S. Moderated estimation of fold change and dispersion for RNA-seq data with DESeq2. Genome Biol 2014;15(12):550.
6. Jumper J, Evans R, Pritzel A, et al. Highly accurate protein structure prediction with AlphaFold. Nature 2021;596(7873):583-589.
7. Meng EC, Goddard TD, Pettersen EF, et al. UCSF ChimeraX: Tools for structure building and analysis. Protein Sci 2023;32(11):e4792.
8. Raj B, Irimia M, Braunschweig U, et al. A global regulatory mechanism for activating an exon network required for neurogenesis. Mol Cell 2014;56(1):90-103.
